# Autophagy Inhibition in Trophoblasts Induces Aberrant Shift in CXCR4^+^ Decidual NK Cell Phenotype Leading to Pregnancy Loss

**DOI:** 10.3390/jcm12237491

**Published:** 2023-12-04

**Authors:** Nan Liu, Huihui Shen, Zehua Wang, Xueyun Qin, Mingqing Li, Xinyan Zhang

**Affiliations:** 1 Laboratory for Reproductive Immunology, Hospital of Obstetrics and Gynecology, Fudan University, Shanghai 200080, China; 2 Obstetrics and Gynecology Hospital of Fudan University, Shanghai 200080, China; 3 Shanghai Key Laboratory of Female Reproductive Endocrine Related Diseases, Hospital of Obstetrics and Gynecology, Fudan University, Shanghai 200080, China

**Keywords:** NK cells, trophoblast, autophagy, IGF2, pregnancy loss, CXCR4

## Abstract

Background: Pregnancy, a complex biological phenomenon, relies on intricate maternal–fetal interactions for success. Decidual natural killer (dNK) cells and trophoblasts are pivotal in establishing immune tolerance at the maternal–fetal interface. The chemokine receptor CXCR4 plays a crucial role in NK cell development and immune tolerance during early placental development. Methods: Primary decidual immune cells from 42 women with normal pregnancies and 20 patients experiencing recurrent spontaneous abortions (RSAs) were studied. Gene transcription in NK cells was assessed using real-time polymerase chain reaction. In a co-culture system, we examined the influence of trophoblasts on CXCR4 expression in dNK cells, with subsequent analysis conducted via flow cytometry. The proportion of CXCR4^+^ NK cells was assessed using flow cytometry after co-culture with trophoblasts pre-treated with 3-MA or a p53 activator. Results: Our study confirmed a diminished presence of decidual CXCR4^+^ NK cells in RSA patients during early pregnancy. Co-culturing with a trophoblast-derived supernatant increased CXCR4 expression in dNK cells. In addition, trophoblast autophagy plays an educative role in regulating the dNK landscape via the IGF2-TP53-CXCR4 axis. Conclusion: Autophagy inhibition in trophoblasts induces an aberrant shift in the CXCR4^+^ dNK phenotype, potentially contributing to pregnancy loss. This sheds light on the nuanced behavior of dNK cells during pregnancy, offering promising therapeutic avenues to mitigate pregnancy complications.

## 1. Introduction

The maternal–fetal interface serves as the epicenter for the intricate dance of molecular and cellular interactions that dictate the success or failure of a pregnancy [1]. This delicate balance can be disrupted by a breakdown in maternal–fetal immune tolerance, potentially culminating in abnormal pregnancies, including recurrent spontaneous abortion (RSA) [2]. Characterized by two or more consecutive pregnancy losses prior to 20 weeks, RSA affects approximately 1–3% of women, thus constituting a formidable challenge in the field of reproductive medicine [3]. The enigma of RSA has long been a vexing challenge in the realm of reproductive health [4]. Deciphering the immunological changes triggered by pregnancy holds the potential to not only open new therapeutic pathways for improving pregnancy outcomes but also offer fresh insights into the broader mechanisms governing immune tolerance, with potential implications for diverse physiological and pathological contexts.

Numerous mechanisms have been postulated to underlie immune tolerance at the maternal–fetal interface during early pregnancy [5,6], encompassing the roles of decidual macrophages, Foxp3^+^ regulatory T cells, and CXCR4^+^ NK cells [1,6,7]. Previous studies have reported that patients with RSAs exhibited insufficient autophagy in decidual stromal cells and reduced residence of dNK cells. Furthermore, mice subjected to NK cell depletion exhibit notable deficiencies in placental vascular remodeling, reduced rates of implantation, and a heightened incidence of embryo loss [8]. Our recent investigations unveiled a noteworthy link between unexplained RSAs and deficient autophagy and cellular residence of decidual macrophages. Mechanistically, a disrupted lysophosphatidic acid–autophagy axis has been implicated in elevating the risk of miscarriage by limiting the residence of these decidual macrophages [6].

dNK cells, representing the most abundant immune cell population at the maternal–fetal interface, play a pivotal role in processes like decidualization, angiogenesis, and embryo implantation [8]. During human pregnancy, the prevalence of dNK cells typically surges to approximately 70% between the 9th and 12th weeks. In contrast, macrophages and T lymphocytes each account for approximately 10–20% of the cellular composition during this gestational period [9]. These dNK cells, distinct from conventional circulating NK cells (CD16^+^CD56^dim^), play a central role in fostering immune tolerance to the fetus. They further engage in facilitating extravillous trophoblast (EVT) cell invasion and participate in the remodeling of uterine spiral arteries via interactions with EVT cells at the maternal–fetal interface [10]. Notably, it is also hypothesized that trophoblasts are instrumental in recruiting and reprogramming peripheral NK cells into CXCR4^+^CD56^bright^ dNK cells during early pregnancy [7].

CXCR4 emerges as a critical regulator governing the development of NK cells and dendritic cells, both of which assume pivotal roles in early placental development and the maintenance of immune tolerance at the maternal–fetal interface [7]. In murine models lacking CXCR4, a decrease in dNK cell numbers is observed, coupled with abnormal formation of abnormal dNK cell aggregates and the infiltration of NK cells into trophoblast areas beyond the giant cell layer. Additionally, these changes are concomitant with diminished NK cell expression of granzyme B, a crucial effector molecule indicative of impaired NK cell function [11]. Importantly, a reduced proportion of CXCR4^+^ NK cells is not only documented in patients with recurrent implantation failure [12] but also in those experiencing RSAs [7].

In light of these complex immunological interactions, it is clear that the interplay between dNK cells and trophoblasts at the maternal–fetal interface represents a multifaceted and indispensable facet of reproductive biology. However, substantial gaps persist in our comprehension of the precise nature of the communication between CXCR4^+^ dNK cells and trophoblasts. Thus, this study was undertaken with the goal of elucidating the mechanisms underpinning the decrease in CXCR4^+^ NK cell numbers observed in the context of RSA.

## 2. Materials and Methods

### 2.1. Participants and Tissue Samples

This study was granted approval by the Ethics Committee of the Obstetrics and Gynecology Hospital of Fudan University (approval number: kyy2019-94) and secured informed consent from all participants before the tissue sample collection. Decidual tissue samples from the first trimester (7–9 weeks of gestation; *n* = 42) were obtained from normal pregnant women undergoing surgical termination for nonmedical reasons. For the RSA group, all subjects (7–9 weeks of gestation; *n* = 20) had regular menstrual cycles and a history of two or more miscarriages with the same partner before 20 weeks, excluding cases with endocrine, anatomic, and genetic abnormalities, as well as infections. Surgical specimens, gathered under aseptic conditions, were promptly immersed in DMEM/F-12 (Hyclone) medium with 1% penicillin–streptomycin (NCM Biotech, Suzhou, China; C125C8). After being transported to the laboratory on ice within 30 min, the samples were processed for dNK cell isolation.

### 2.2. Cell Isolation and Culture

The decidual tissues underwent meticulous rinsing with PBS (Servicebio, Wuhan, China) and were minced to 1 mm^3^ fragments prior to digestion with a 0.1% solution of type IV collagenase (Worthington, Lakewood, NJ, USA; LSO04186) for 30 min at 37 °C. The ensuing process involved filtration through 100-, 200-, and 400-mesh filters, followed by centrifugation at 1500 rpm for 10 min to discard the supernatant. Decidual immune cells (DICs) were isolated using density gradient centrifugation through 20%, 40%, and 60% Percoll (Amersham, Marlborough, MA, USA), and then the lower layer DICs were recovered after centrifugation at 2500 rpm for 30 min. According to the manufacturer’s instructions (MiltenyiBiotec, Bergisch Gladbach, North Rhine-Westphalia, Germany), dNK cells were separately obtained from DICs via negative selection using the human NK cell magnetic activated cell sorting (MACS) kit and verified via flow cytometry. The isolated NK cells were cultured in RPMI-1640 medium (Gibco, Grand Island, NY, USA) supplemented with 10% FBS (Gibco, Grand Island, NY, USA) and 1% penicillin–streptomycin (NCM Biotech, Suzhou, China). The culture conditions were varied, with or without the inclusion of IGF2 (50 ng/mL; PeproTech, Cranbury, NJ, USA; 100-12-10UG) and NSC59984 (12 μM; Selleck, Houston, TX, USA; S8106).

### 2.3. Co-Culture System

Preceding co-cultivation with NK-92 cells (NK cell line), HTR-8/SVneo cells underwent a 48 h pre-treatment with or without 3-MA (10 mM; Sigma, St. Louis, MO, USA) and NSC59984 (12 μM; Selleck, Houston, TX, USA; S8106). After pre-treatment, conditioned medium from the HTR-8/SVneo cultures was harvested and utilized for the indirect co-culture with dNK or NK-92 cells. After a co-culture duration of 48 h, all cells suspended in the co-culture system were collected for ensuing experiments.

### 2.4. Flow Cytometry Assays

Fluorochrome-conjugated antibodies for the following human antigens were used for flow cytometry analysis: anti-human CD45 (Brilliant Violet 605™, Biolegend, San Diego, CA, USA; 368523), anti-human CD3 (Brilliant Violet 510™, Biolegend, San Diego, CA, USA; 317331), CD56 (PE, Biolegend, San Diego, CA, USA; 985902), and anti-human (CD184) CXCR4 (APC, Biolegend, San Diego, CA, USA; 306509). Surface staining was performed at 25 °C for 30 min in a 100 μL reaction volume, shielded from light. After adequate washing, the labeled cells were suspended in 500 μL of Cell Staining Buffer and subjected to flow cytometry analysis using a CytoFLEX flow cytometer (Beckman Coulter, Brea, CA, USA) with CytExpert software (Version 2.4). Data processing and analysis were performed using FlowJo software (Version 10.8.1, Ashland, OR, USA).

### 2.5. Quantitative Reverse Transcription PCR (qRT-PCR)

Total RNA extracted from diverse cell samples subjected to various treatments was accomplished using the EZ-press RNA Purification Kit (EZBioscience, Roseville, MN, USA; B0004DP). Subsequent to this extraction, reverse transcription into cDNA was conducted utilizing the PrimerScript™ RT reagent kit (Takara, Shiga, Japan; RR047A). Amplification reactions were executed using a ChamQ SYBR Color qPCR Master Mix (Vazyme, Nanjing, China; Q411), and analyses were carried out with the QuantStudio™ 6 Flex Real-Time PCR System (Thermo Fisher Scientific, Waltham, MA, USA).

The fold changes in the transcriptional levels of target genes were calculated using the 2^−ΔΔCt^ method, with each sample run in triplicate. mRNA expression values were normalized against the corresponding β-actin (*ACTB*) values. The primer sequences for target genes can be found in Table 1.

### 2.6. Bioinformatics Analysis

RNA sequencing (RNA-seq) was performed by Shanghai Litzchi Biosystems (LITCHI BIO, Shanghai, China), and data analysis was conducted as described in our previous study [13].

The forecast of signaling network interactions involving IGF2 and the chemokine receptor CXCR4 was conducted utilizing the STRING database (https://string-db.org/, accessed on 25 September 2023). Following the retrieval of subsequent data, visualization was achieved using Cytoscape (Version 3.7.1; San Diego, CA, USA).

### 2.7. Statistical Analysis

Data are presented as means ± SD. For comparisons between two groups, the unpaired t-test was utilized. Statistically significant differences among three or more groups were determined using one-way ANOVA, followed by a Bonferroni test for multiple comparisons. Analysis and graphing were performed using GraphPad Prism software (Version 9.4.1; San Diego, CA, USA). A *p*-value of <0.05 was considered statistically significant, with significance denoted as follows: * for *p* < 0.05, ** for *p* < 0.01, *** for *p* < 0.001, and **** for *p* < 0.0001.

## 3. Results

### 3.1. Reduced CXCR4 Expression in dNK Cells of RSA Patients

**Figure 1:**
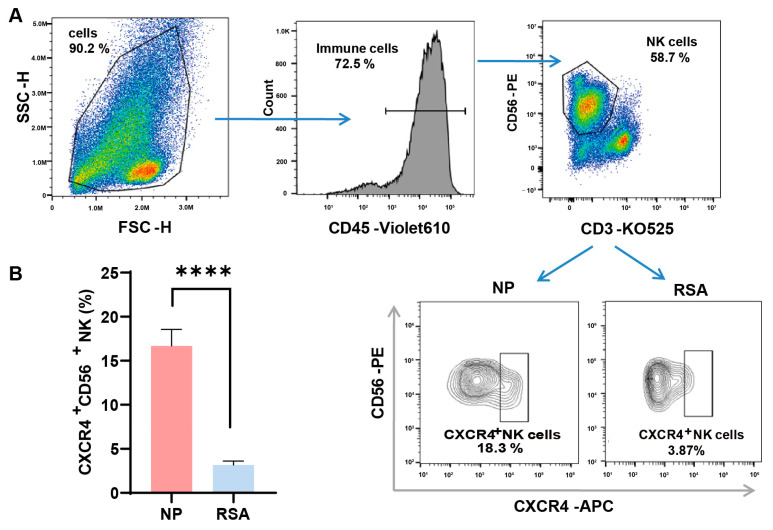
Diminished CXCR4 expression in decidual NK cells of RSA patients. (**A**) Detailed gating strategies employed in the examination of CXCR4 expression in decidual natural killer (dNK) cells using flow cytometry. (**B**) Quantitative analysis of the proportion of CXCR4^+^ dNK cells in the recurrent spontaneous abortion (RSA) group (*n* = 10) compared with the normal pregnancy (NP) group (*n* = 10). Data are presented as means ± SD, with statistical significance indicated. **** *p* < 0.0001.

We first examined the expression of CXCR4 on the surfaces of dNK cells by employing flow cytometry and compared their expression in the decidual tissue between RSA patients and controls. The detailed gate strategies are shown in Figure 1A. Upon gating the CD3-CD56^+^ NK cell subset, a marked reduction in the proportion of CXCR4^+^ dNK cells was evident in the RSA group in comparison with the normal pregnancy group. Subsequent quantitative analysis confirmed a statistically significant variance between the two groups (Figure 1B).

### 3.2. Enhanced CXCR4 Expression in dNK Cells Co-Cultured with Trophoblast-Derived Supernatant

**Figure 2:**
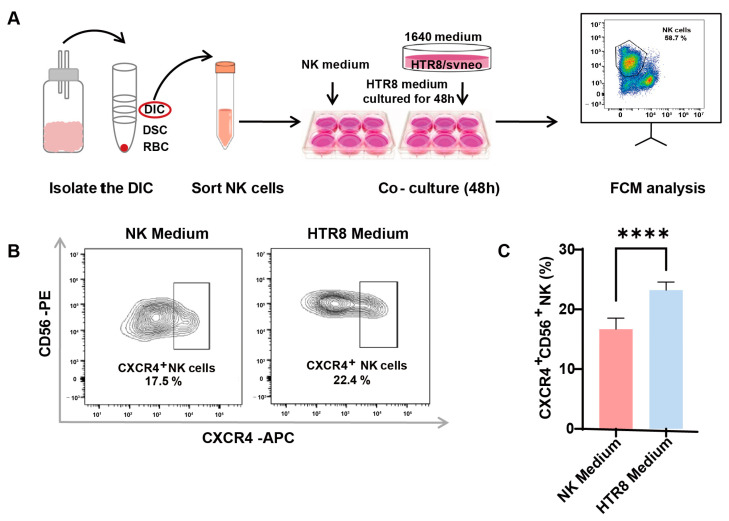
Enhanced CXCR4 expression in dNK cells co-cultured with trophoblast-derived supernatant. (**A**) The schematic diagram of decidual lymphocyte isolation and subsequent sortation, as well as the establishment of an indirect co-culture system. (**B**) Representative flow cytometry graphs of CXCR4 expression in dNK cells exposed to different culture environments. (**C**) Quantitative analysis of the proportion of CXCR4^+^CD56^+^ dNK cells when co-cultured with HTR8 medium compared with NK medium (*n* = 6). Data are presented as means ± SD. **** *p* < 0.0001.

The involvement of trophoblasts in recruiting and modulating peripheral NK cells into CXCR4^+^CD56^bright^ dNK cells during early pregnancy has been documented (7). To ascertain whether autophagy in trophoblasts influences CXCR4 expression in dNK cells via paracrine signaling, primary dNK cells were assessed following co-culture with or without a trophoblast supernatant (HTR8/SVneo cell line, a trophoblast cell line) (Figure 2A). After the successful isolation of primary dNK cells, we examined CXCR4 expression in dNK cells using flow cytometry (Figure 2B). Notably, our findings revealed a significant increase in CXCR4^+^CD56^+^ NK cells when dNK cells were exposed to the trophoblast-derived supernatant, as opposed to those maintained in the NK medium (Figure 2C). This substantial elevation in CXCR4 expression within the HTR8 medium setting underscores the potential regulatory influence of trophoblast-derived components on the behavior of dNK cells. 

These results suggest that trophoblast-derived elements present in the HTR8 medium may play a pivotal role in shaping the phenotype and function of dNK cells, potentially influencing pregnancy outcomes. This realization serves as a compelling impetus for further investigations aimed at uncovering the underlying mechanisms at play in this context.

### 3.3. Autophagy Inhibition in Trophoblasts Restricts CXCR4 Expression in dNK Cells

**Figure 3:**
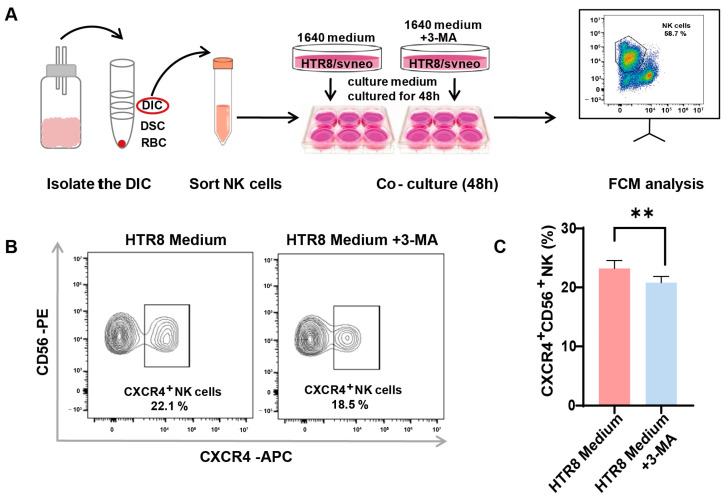
Autophagy inhibition in trophoblasts restricts CXCR4 expression in dNK cells. (**A**) The schematic depicts the process of isolating decidual natural killer (dNK) cells and establishing an indirect co-culture system using both conventional and 3-MA pre-treated HTR8/SVneo conditioned mediums. (**B**) Representative flow cytometry graphs display the variation in CXCR4 expression in dNK cells subjected to different culture environments (*n* = 6). (**C**) Quantitative analysis demonstrates the proportion of CXCR4^+^CD56^+^ NK cells when co-cultured with 3-MA pre-treated HTR8 medium compared with the untreated condition, with data expressed as means ± SD. ** *p* < 0.01.

A preceding study documented a diminished level of autophagy in trophoblasts among patients with RSAs and underscored that autophagy in trophoblasts may constrain the cytotoxicity of dNK cells [13]. In our endeavor to unravel the intricate association between autophagy and its effects on CXCR4 expression in dNK cells, we instituted an indirect co-culture system. Following the meticulous isolation of dNK cells from decidual tissue and their subsequent co-cultivation (Figure 3A), we employed flow cytometry to assess CXCR4 expression. Our observations revealed a significant reduction in the population of CXCR4^+^CD56^+^ NK cells in the presence of 3-methyladenine (3-MA), an autophagy inhibitor (Figure 3B). This pronounced difference in CXCR4 expression was subsequently validated via quantitative analysis (Figure 3C).

This intriguing revelation suggests the potential involvement of trophoblast cell autophagy in regulating CXCR4 expression within dNK cells, thus paving the way for further inquiries into the mechanistic pathways that underlie the intricate interplay between trophoblast autophagy and the modulation of immune responses within the decidual microenvironment. 

### 3.4. Trophoblast Autophagy Modulates CXCR4 Expression in NK-92 Cells via IGF2

**Figure 4:**
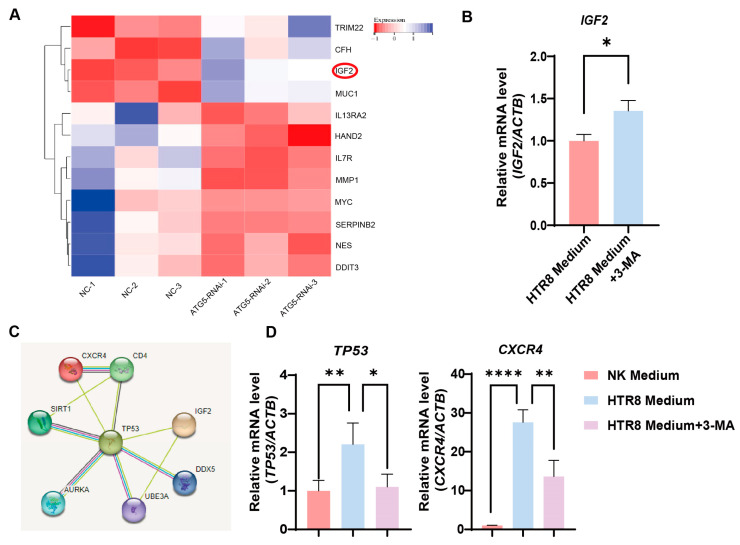
Trophoblast autophagy modulates CXCR4 expression in dNK cells via IGF2. (**A**) Heatmap of differentially expressed genes closely related to NK function upon trophoblast autophagy inhibition (*n* = 3). (**B**) The *IGF2* mRNA expression in trophoblast cells subjected to different culture environments (*n* = 6). (**C**) The predicted networks obtained from the STRING database. (**D**) The *TP53* and *CXCR4* mRNA expression in NK-92 cells (NK cell line) subjected to different culture environments (*n* = 6). Data are presented as means ± SD. * *p* < 0.05, ** *p* < 0.01, and **** *p* < 0.0001.

To delineate the molecular dynamics governing the influence of trophoblast autophagy on CXCR4^+^ dNK cells, we conducted a bioinformatics analysis. Importantly, the differential gene heatmaps identified insulin-like growth factor 2 (IGF2) as a central gene showing upregulation subsequent to the suppression of autophagy in trophoblast cells (Figure 4A). This upregulation was further substantiated by employing RT-PCR to measure the augmented levels of IGF2 mRNA in autophagy-inhibited conditions (Figure 4B). This finding, in turn, steered our focus toward the plausible IGF2-mediated regulatory networks that might be influencing NK-92 cells. As a result of our rigorous investigations, *TP53* (its protein name is P53, a tumor suppressor protein) emerged as a compelling candidate to bridge the gap between IGF2 and the regulation of CXCR4, as substantiated by the STRING database (Figure 4C). Consistently, when we co-cultured NK-92 cells with the trophoblasts, an upregulation in *TP53* and *CXCR4* expression was observed, whereas the application of 3-MA pre-treatment resulted in a marked reduction in their expression (Figure 4D). Therefore, trophoblast autophagy modulates CXCR4 expression in NK cells, potentially acting via IGF2-TP53 signaling, and orchestrates the behavior of NK cells by modulating the expression of CXCR4. 

### 3.5. Trophoblast Autophagy Influences NK Cells via IGF2-TP53-CXCR4 Axis

**Figure 5:**
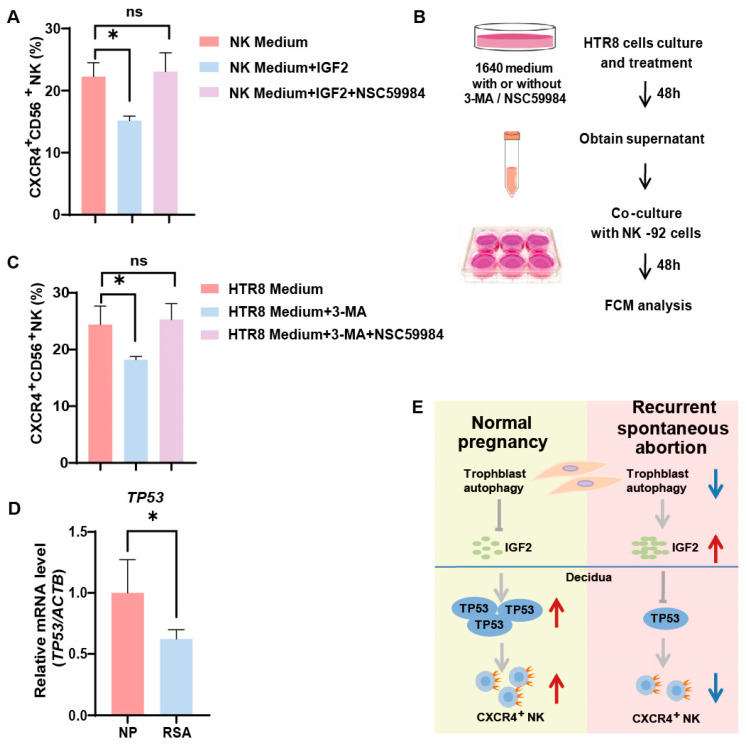
Trophoblast autophagy influences dNK cells via IGF2-TP53-CXCR4 axis. (**A**) The percentage of CXCR4^+^CD56^+^ NK cells subjected to different culture environments and treated with *IGF2* (50 ng/mL; 48 h) and a P53 pathway activator, NSC59984 (12 μM; 48 h) (*n* = 6). (**B**) The schematic depicts the process of trophoblast (HTR8) treatment and the establishment of an indirect co-culture system with NK-92 cells. (**C**) The percentage of CXCR4^+^CD56^+^ NK cells subjected to different culture environments and treated with 3-MA (10 mM; 48 h) and a P53 pathway activator, NSC59984 (12 μM; 48 h) (*n* = 6). (**D**) The mRNA expression of *TP53* in decidual tissues from normal pregnancy (NP) group (*n* =10) and recurrent spontaneous abortion (RSA) group (*n* = 10). ns, not significant; * *p* < 0.05. (**E**) A schematic overview that encapsulates the interconnected relationships between the IGF2-TP53-CXCR4 axis and trophoblast autophagy. Red arrows indicate promotion or up-regulation, while blue arrows denote inhibition or down-regulation.

Our previous research unveiled a decrease in autophagy levels and *IGF2* expression in villi among patients with RSAs [13]. From the aforementioned results, it became evident that trophoblast autophagy had the potential to influence *CXCR4* expression in NK cells via *IGF2*. Furthermore, we identified that *IGF2*, potentially acting in conjunction with *TP53*, effectively orchestrates the behavior of NK-92 cells by modulating the expression of *CXCR4*. In the in vitro NK-92 cell line, we activated p53 in NK cells using NSC59984 (a p53 activator) and performed flow cytometry. The addition of IGF2 partially reversed the positive effect of trophoblast autophagy on CXCR4 expression within NK cells (Figure 5A). While the activation of p53 significantly upregulated CXCR4 expression in NK cells (Figure 5A). To elucidate the complex interplay between autophagy and *TP53* expression in the regulation of *CXCR4* expression in NK cells, we established an indirect co-culture system. HTR8 cells were pre-treated with or without 3-MA or NSC59984 for 48 h, followed by the collection of the supernatant for indirect co-culture with NK-92 cells for another 48 h. Subsequently, the expression of CXCR4 was assessed using flow cytometry (Figure 5B). As demonstrated in Figure 5C, in the indirect co-culture system with HTR8/SVneo cells, the percentage of CXCR4^+^ NK cells witnessed a significant decrease in the 3-MA-treated group, in alignment with our previous findings. Furthermore, the addition of NSC59984 had the capacity to reverse this downregulation (Figure 5C). In addition, the mRNA levels of *TP53* were significantly reduced in the dNK cells of RSA patients (Figure 5D). Collectively, these data provide compelling validation of the proposition that trophoblast autophagy possesses the capacity to influence dNK cells toward an immunomodulatory phenotype via the IGF2-TP53-CXCR4 axis (Figure 5E). 

## 4. Discussion

A previous study reported that CXCR4^+^CD56^bright^ dNK cells can be recruited from the peripheral blood and be reprogrammed by trophoblasts, thus actively participating in the establishment of immune tolerance during early pregnancy [7]. A fundamental observation that emerges from this study is the diminished presence of decidual CXCR4^+^ NK cells during the early stages of pregnancy in RSA subjects, suggesting their deficit can disrupt the equilibrium at the maternal–fetal interface. This is consistent with previous findings that CXCR4 expression in dNK cells was decreased in both miscarriage patients and abortion-prone mice [7]. Given that CXCR4 dNK cells have great therapeutic potential in the treatment of pregnancy failures such as miscarriages [7], it is important to unveil the mechanisms underpinning the decrease in CXCR4^+^ NK cell number observed in the context of RSA.

The orchestration of the maternal–fetal interface is a symphony where every note must be played precisely. In the context of this study, trophoblast autophagy emerged as a crescendo that modulates the dynamics of CXCR4 expression. The underlying mechanisms that regulate this decline in the CXCR4^+^ NK cell number were unveiled through an in-depth exploration of the interaction between dNK cells and trophoblasts. 

Emerging evidence suggests a correlation between RSA and altered trophoblast cell behavior, particularly insufficient trophoblast invasion [14]. Autophagy, a crucial process for energy production, plays an indispensable role in successful pregnancies [6,8,15]. Tan et al. observed significantly lower autophagy activity in the villi of RSA patients compared with elective pregnancy termination patients using transmission electron microscopy, highlighting the role of autophagy in pregnancy outcomes [13]. Chen et al. also documented reduced autophagy levels in the trophoblast cells of RSA patients compared with normal pregnant women via Western blot and RT-PCR analysis [16]. A recent study confirmed diminished protein levels of autophagy-related molecules, including Beclin-1 and LC3II/I, in villus tissues from missed abortion patients, as well as autophagosomes [17]. These findings collectively suggest that inadequate trophoblast autophagy might be a key factor in RSA occurrence. 

Tan’s research linked impaired trophoblast cell autophagy to an elevation in IGF2 expression [13]. Our study revealed that the resulting increase in the IGF2 levels of trophoblasts, in turn, exerts a discernible influence on TP53 activation in dNK cells, substantiating the interplay between trophoblast autophagy and CXCR4^+^ dNK cells. This intricate cascade, with its far-reaching implications, culminates in the transformation of dNK cells, leading to a shift toward a CXCR4-negative phenotype. The inhibitory effect of IGF2 on CXCR4 expression is potentiated by P53 activation, indicating the presence of a regulatory loop that influences dNK cell behavior. Tan et al. verified that inhibiting autophagy in trophoblasts enhances the killing activity of dNK cells by increasing IGF2 secretion and increased fetal loss in mice [13]. While Tao’s work reported the involvement of dysfunctional CXCR4^+^ dNK cells in the Th1 response during miscarriages [7], our study, in contrast, focused on the impact of trophoblast autophagy on CXCR4^+^ dNK cells, bridging a gap in the existing research. 

IGF2 is one of the most potent embryonic growth factors secreted mainly by trophoblasts and has a pivotal role in regulating the expansion of the placental vascular tree during late gestation [18,19]. Excessive IGF2 expression has often been observed in tumor patients [20]. Meanwhile, TP53, a crucial housekeeping gene, exerts its influence over a range of cellular processes, including apoptosis, senescence, differentiation, autophagy, and metabolism [21]. Moreover, cAMP-induced decidualization leads to a substantial upregulation of p53, driven by protein stabilization [22]. This is underscored by the developmental lethality observed in p53-homozygote-null mice that lack the paternally expressed allele of imprinted Igf2 [23]. In concordance with this knowledge, our findings reveal a distinct decrease in TP53 and IGF2 expression in RSA patients, a downregulation that can be attributed to trophoblast autophagy. Subsequently, this impairment in the IGF2-TP53 signaling axis has a profound impact on the dNK cell phenotype, leading to a decrease in CXCR4 expression in RSA patients. These discoveries open new avenues for potential therapeutic approaches to address the complexities of RSA. 

This intricate comprehension of cellular interactions underlying RSA not only enriches our understanding of the condition but also reveals promising avenues for diagnosis and therapy. More specifically, a previous study reported that CXCR4^+^ dNK cells originated from pNK cells recruited and reprogrammed by trophoblasts [7]. Future investigations could delve into whether the reduction in CXCR4^+^ NK cells in the peripheral blood is associated with the decrease in CXCR4^+^ dNK cells at the maternal–fetal interface and their causal relationship with RSA. If a robust correlation is established, CXCR4^+^ NK cells may emerge as a potential early warning marker for RSA. In addition, Tao et al. discovered that the embryo resorption rate in recipient Nfil3^−/−^ mice was significantly reduced via the adoptive transfer of CXCR4^+^ dNK cells, whereas CXCR4^−^ dNK cells did not exhibit the same effect [7]. This indicates the promising potential of CXCR4^+^ dNK cells as a cell-based immunotherapy for pregnancy failure. Likewise, modulating the influence of dNK cells by regulating trophoblast cell autophagy levels and the IGF2-P53-CXCR4 axis are anticipated to hold interventional therapeutic value for RSA. 

These in vitro insights, albeit illuminating, are punctuated by limitations, such as the use of the HTR8/SVneo cell line, which may not faithfully mirror primary trophoblast behavior and, therefore, warrants cautious interpretation. Moreover, the absence of in vivo corroborations signals a crucial nexus for future explorations, necessitating nuanced investigations into the physiological relevance and therapeutic applicability of the IGF2-TP53-CXCR4 axis in managing complex pregnancy outcomes, potentially offering a mechanistic bridge from cellular crosstalk to clinical paradigms. Future research endeavors will prioritize the expansion of the sample size to enhance the robustness of our findings, facilitating more reliable and generalizable outcomes. 

In conclusion, our study uncovered the role of trophoblast autophagy in regulating CXCR4 expression in dNK cells via the IGF2-TP53-CXCR4 axis, shedding light on the intricate dynamics at the maternal–fetal interface. These findings contribute to our understanding of dNK cell behavior in pregnancy and open doors to potential therapeutic interventions for pregnancy complications.

## Figures and Tables

**Table 1:** Gene primers for qRT-PCR.

*IGF2*	Forward	CAATGGGGAAGTCGATGCTG
(human)	Reverse	GGAAACAGCACTCCTCAACG
*TP53*	Forward	AGGTTGGCTCTGACTGTACC
(human)	Reverse	TCTTCTTTGGCTGGGGAGAG
*CXCR4*	Forward	TGTCATCACGCTTCCCTTCT
(human)	Reverse	TCATCTGCCTCACTGACGTT
*ACTB*	Forward	GGCATCCTCACCCTGAAGTA
(human)	Reverse	TAGCACAGCCTGGATAGCAA

## Data Availability

Data are contained within the article.

## Abbreviations

NP	Normal pregnancy
RSA	Recurrent spontaneous abortion
IGF2	Insulin-like growth factor 2
3-MA	3-methyladenine
DIC	Decidual immune cell
RBC	Red blood cell
FCM	Flow cytometry
